# Developmental programming of the neuroendocrine axis by steroid hormones: Insights from the sheep model of PCOS

**DOI:** 10.3389/fendo.2023.1096187

**Published:** 2023-01-23

**Authors:** Sara Gurule, Jessica Sustaita-Monroe, Vasantha Padmanabhan, Rodolfo Cardoso

**Affiliations:** ^1^ Department of Animal Science, Texas A&M University, College Station, TX, United States; ^2^ Department of Pediatrics, University of Michigan, Ann Arbor, MI, United States

**Keywords:** androgens, hypothalamus, pituitary, PCOS (polycystic ovarian syndrome), sheep

## Abstract

The reproductive neuroendocrine system is a key target for the developmental programming effects of steroid hormones during early life. While gonadal steroids play an important role in controlling the physiological development of the neuroendocrine axis, human fetuses are susceptible to adverse programming due to exposure to endocrine disrupting chemicals with steroidal activity, inadvertent use of contraceptive pills during pregnancy, as well as from disease states that result in abnormal steroid production. Animal models provide an unparalleled resource to understand the effects of steroid hormones on the development of the neuroendocrine axis and their role on the developmental origins of health and disease. In female sheep, exposure to testosterone (T) excess during fetal development results in an array of reproductive disorders that recapitulate those seen in women with polycystic ovary syndrome (PCOS), including disrupted neuroendocrine feedback mechanisms, increased pituitary responsiveness to gonadotropin-releasing hormone (GnRH), luteinizing hormone (LH) hypersecretion, functional hyperandrogenism, multifollicular ovarian morphology, and premature reproductive failure. Similar to a large proportion of women with PCOS, these prenatally T-treated sheep also manifest insulin resistance and cardiovascular alterations, including hypertension. This review article focuses on the effects of prenatal androgens on the developmental programming of hypothalamic and pituitary alterations in the sheep model of PCOS phenotype, centering specifically on key neurons, neuropeptides, and regulatory pathways controlling GnRH and LH secretion. Insights obtained from the sheep model as well as other animal models of perinatal androgen excess can have important translational relevance to treat and prevent neuroendocrine dysfunction in women with PCOS and other fertility disorders.

## 1 Introduction

### 1.1 Polycystic ovary syndrome (PCOS) and neuroendocrine dysfunction

Approximately 60 to 80 million people experience difficulty conceiving globally (1) and in 30 to 40% of couples of childbearing age seeking fertility counseling, infertility is exclusively a problem with the female. Polycystic ovary syndrome (PCOS) is the most common infertility disorder affecting approximately 5 million women in the U.S. and over 100 million worldwide (2). PCOS is characterized by reproductive alterations including oligo-/anovulation, increased tonic secretion of luteinizing hormone (LH), and hyperandrogenism (3). Moreover, approximately 70% of women with PCOS manifest metabolic imbalances, such as obesity and insulin resistance (4). Despite the high prevalence of PCOS globally, the origins, causes, and pathophysiology of this syndrome remain largely unknown. Epidemiological data suggest that neuroendocrine alterations, such as increased pulsatile secretion of gonadotropin-releasing hormone (GnRH), enhanced pituitary sensitivity to GnRH stimulation, and resulting LH hypersecretion are generally observed in PCOS patients and likely contribute to its etiology (5). The rapid pulsatile release of GnRH favors LH synthesis and secretion over follicle-stimulating hormone (FSH). The increased pulse frequency of LH, in turn, stimulates theca cells to synthesize more androgens, while the relative low levels of FSH reduce the capacity of granulosa cells to aromatize androgens, resulting in the hyperandrogenic condition. On the flip side, elevated concentrations of androgens impair the responsiveness of the neuroendocrine system to the progesterone and estradiol negative feedback mechanisms on tonic secretion of GnRH and LH, thus creating a vicious circle between LH hypersecretion and hyperandrogenism (5, 6). In most PCOS women, higher estradiol and progesterone doses are required to reduce LH pulse frequency (7, 8). Moreover, while testosterone administration does not increase LH pulse frequency in women (9), androgen antagonist therapy re-establishes the ability of estradiol and progesterone to suppress LH pulsatility in PCOS patients (10), suggesting that hyperandrogenism does not directly drive LH hypersecretion, but instead it reduces the ability of ovarian steroids to suppress LH. Clinical studies indicate that these neuroendocrine defects develop early in life since hyperandrogenic girls exhibit elevated pulsatile secretion of LH before menarche (11, 12). Additionally, PCOS women exhibit a greater LH response to exogenous GnRH stimulation (13–15), implying a role for the anterior pituitary gland in the LH hypersecretion. While alterations in the secretory pattern of LH have been characterized extensively in women with PCOS, for obvious reasons, tissue-specific processes associated with LH hypersecretion are difficult to be determined; therefore, animal models of PCOS provide unparalleled resources to investigate cellular and molecular mechanisms at the hypothalamo-pituitary axis that underlie the neuroendocrine dysfunction.

### 1.2 Developmental origins of PCOS and epigenetic modifications

Epidemiological data and preclinical studies indicate that several factors including genetics, epigenetics, and environmental conditions are involved in the pathogenesis of PCOS. Genome-wide association studies (GWAS) found numerous susceptibility loci in women with PCOS, however the currently known loci only explain less than 10% of PCOS’ heritability (16, 17), thus indicating that other factors such as *in utero* environmental conditions *via* epigenetic modifications may account for the remaining heritability. Prenatal exposure to androgen excess is the environmental insult most widely associated with the development of PCOS traits. Preclinical and clinical studies clearly show that elevated intrauterine exposure to androgens increases the risks of the female offspring to develop the PCOS phenotype later in life (18, 19). Daughters of women with congenital adrenal hyperplasia, a condition in which the adrenal cortex produces abnormally high amounts of androgens, have a significantly greater risk of developing PCOS (20). Likewise, daughters of women with PCOS have a higher likelihood of developing PCOS-like reproductive and metabolic alterations (21–23), further suggesting that abnormal levels of prenatal androgens can impair the development of the female offspring. Given the crucial effects of epigenetic mechanisms in the fetal origins of adult diseases (24, 25), the involvement of epigenetic processes, such as DNA methylation, in the etiology of PCOS was recently explored. Differential DNA methylation and gene expression profiles have been reported in ovarian tissues from women with PCOS, including changes within pathways related to the pathogenesis of PCOS (26, 27). Epigenetic studies in adipose tissue from women with PCOS also identified a large number of differently expressed genes with corresponding changes in DNA methylation patterns (28). Importantly, findings that global DNA methylation in peripheral blood leukocytes is unaltered in PCOS patients versus matched controls (29) emphasize the need for site-specific epigenetic studies in physiologically-relevant target tissues. Since neuroendocrine tissues from women are nearly unattainable, epigenetic alterations in the hypothalamus or pituitary from women with PCOS have not been explored, highlighting the importance of PCOS animal models.

### 1.3 Sheep model of PCOS phenotype

Animal models of PCOS phenotype represent a valuable tool to identify the pathophysiological mechanisms associated with the development and manifestation of PCOS traits. Among the several animal models developed, rodents, sheep, and rhesus monkeys are the most well characterized in PCOS research with each model presenting different benefits and limitations (30–34). The rhesus monkey remains the best model for translational relevance and similarity to humans. However, the rhesus monkey has a long reproductive developmental timeline, reaching reproductive competence between 2.5 and 3.5 years of age (35), compared to sheep who reach puberty between 28 to 33 weeks of age (36). The rhesus monkey is also economically difficult to maintain, limiting their research usage. Rodents are able to reproduce rapidly and are cost efficient compared to both the sheep and monkey models. However, due to their small size, repetitive blood sampling and hormonal profiling is difficult, and the translational relevance to humans is limited. Disadvantages of the sheep model include having a synepitheliochorial placenta rather than hemochorial and the fact that sheep are not as genetically similar to humans as the rhesus monkey. Though, since sheep are domesticated animals, they are not exposed to the stressful environments associated with caging (37). Moreover, sheep are considered an important model since their trajectory of organ development is fairly similar to humans (30), and can be used in intensive studies that require repeated sampling and fetal manipulations.

Studies in the female sheep revealed that prenatal treatment with testosterone disrupts the developmental trajectory of the fetus culminating in adult neuroendocrine, ovarian, and metabolic perturbations that closely resemble those seen in women with PCOS (30). Prenatal testosterone-treatment from days 30-90 of gestation (term pregnancy: ~147 days) compromises reproductive function resulting in progressive deterioration of ovarian cyclicity and premature reproductive failure, with most females becoming anovulatory by the second breeding season (early adulthood) (38). In addition, prenatal testosterone-treatment results in intrauterine growth restriction, peripheral insulin resistance, and hypertension in the female sheep (39). Notably, the concentrations of androgens in the fetal circulation achieved with prenatal testosterone treatment in sheep are similar to those reported in male fetuses and are within the physiological range (40, 41). In humans, fetal concentrations of testosterone during the second trimester of gestation are reported to be within the male fetus range in 40% of the female fetuses (42).

Because the use of sheep allows detailed hormonal profiling, previous studies indicate that the progressive reproductive failure seen in prenatal testosterone-treated females stems, at least in part, from tonic activation of the reproductive neuroendocrine axis. Prenatal testosterone-treated sheep present defects in all three steroid feedback mechanisms controlling GnRH and LH secretion, namely estradiol negative (43), estradiol positive (44), and progesterone negative feedback (45, 46). Furthermore, pituitary sensitivity to GnRH is markedly increased in these animals (47). The defects in steroid negative feedback and augmented pituitary responsiveness to GnRH together contribute to the LH excess and consequent functional hyperandrogenism seen in prenatal T-treated sheep.

In this review article, we present a brief overview of the role of prenatal steroid hormones on brain development in sheep, summarize the key neuroendocrine alterations observed in this sheep model of PCOS phenotype, and discuss the potential cellular and molecular mechanisms involved in these neuroendocrine defects. While this review focuses primarily on the sheep model of PCOS-like phenotype, data from other animal models are also included at times for comparative purposes. For description of metabolic and ovarian alterations in prenatally testosterone-treated sheep, we refer readers to the following review articles (30, 39, 48).

## 2 Prenatal steroid hormones and brain development

In mammals, the establishment of a complex hypothalamic neuronal network during early developmental periods is critical for the attainment of reproductive competence during adult life (49). This process involves the development and migration of GnRH neurons as well as the establishment of a complex upstream neuronal network that mediates the feedback regulatory effects of gonadal steroids on both pulsatile and preovulatory surge release of GnRH/LH in females. The organization of this neuronal network occurs early in life in mammals, spanning from late embryonic to early postnatal development (49). Importantly, sexual differentiation of the brain, which is crucial for proper reproductive function and behavior during adulthood, also occurs during this window of perinatal development. Our knowledge regarding the timeline of development of the neuroendocrine axis in sheep remains scarce due to the limited numbers of studies.

In the ovine fetus, the development of the pituitary gland initiates approximately at 19 days of gestation (50, 51). The anterior pituitary develops from Rathke’s pouch where it connects to the infundibulum stalk by day 21 of gestation (51). This is followed by the complete separation of Rathke’s pouch from the epithelial roof of the mouth by day 27 of gestation after both have developed from the endoderm and ectoderm, completing the formation of the anterior pituitary (51). While the anterior pituitary develops from the mouth, the posterior pituitary begins its development from the floor of the brain (50). It starts from a pouch that moves down from the brain to create the infundibulum stalk and ending with the association of both lobes to create the pituitary (50). These processes for development of the anterior and posterior pituitary are completed around day 27 of gestation in sheep (51). Signals from the hypothalamus to the pituitary are relayed through the hypothalamic-pituitary portal system. There is early evidence indicating that this vascular system develops before 45 days of gestation (52). Ovine fetuses within gestational ages of 45 to 67 days were found to have blood vessels projecting from the hypothalamus into the median eminence, infundibulum, pituitary, and pars tubularis (52). Similar evidence was found by Levidiotis et al. (53) after infusing fetal ovine brains with Indian ink. Using brains of younger sheep fetuses at 42 days of gestation the pituitary gland had blood vessels dispersed throughout (53). However, full vascularization is not reached until 45 days of gestation (53).

**Figure 1:**
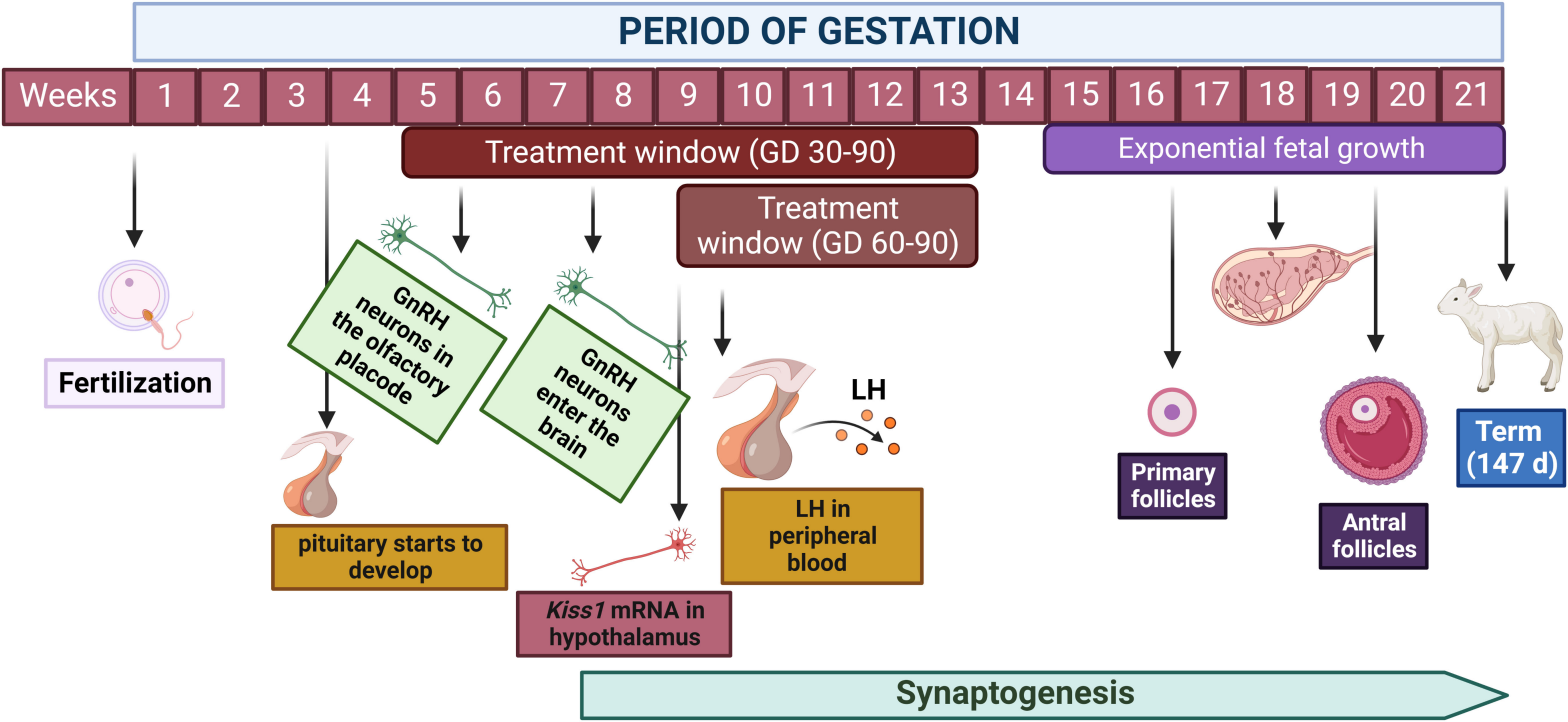
Key events during the developmental ontogeny of the reproductive axis in the ovine fetus. Hypothalamic development and migration of GnRH neurons occur primarily between weeks 5 and 10 of gestation, however, synaptogenesis and neuroplasticity persist throughout gestation and early postnatal life. Pituitary development initiates around day 20 of gestation and LH is detected in the peripheral blood at day 56 of gestation. Primary follicles are present starting on week 17 of gestation while antral follicles develop starting on week 20. This timeline of fetal organogenesis in sheep is developed based on the following references (30, 51, 52, 55, 56). Created with BioRender.com.

After a vascular connection is established between the hypothalamus and the pituitary gland, hypothalamic GnRH neurons can communicate with gonadotrophs in the anterior pituitary to stimulate LH and FSH synthesis and secretion. GnRH neurons first appear in the olfactory placode of the ovine fetus at gestational day 35. Migration into the brain then occurs between embryonic days 49 and 80 (54). LH-containing cells are first detected in the anterior pituitary at 50 days of life in both male and female fetuses. FSH and LH-FSH cells do not appear until 89 days of gestation in male and female fetuses (55). However, research by Roselli et al. (56) has shown the expression of mRNA for FSH β subunit as early as gestational day 59 in the ovine fetal pituitary. Roselli et al. (56) also found evidence of mRNA expression for kisspeptin as early as 59 days of life. As discussed in more detail below, kisspeptin is a potent stimulator of GnRH secretion and a critical peptide mediating the regulatory effects of sexual steroids on GnRH release (57). In the sheep hypothalamus and pituitary, mRNA and protein expression of estrogen receptor-α (ESR1) and estrogen receptor-β (ESR2) appear as early as 80 days of gestation (58). Similar evidence was found for androgen receptors by Wood and Keller-Wood (59). **Figure 1** depicts some key events during prenatal development of the neuroendocrine system in sheep.

Brain developmental time periods are similar between the male and female ovine fetuses, but steroid hormones produced by the fetal gonads create a sexually dimorphic brain. The first use of this term in combination with the idea that steroid hormones are involved in the organization of the brain and behavior was presented in 1980 by Goy and McEwen (60). Goy and McEwen (60) also introduced the idea of a critical period or what they preferred to call a “period of maximal susceptibility”. Early examples of physical, hormonal, and behavioral changes in ewes as a result of prenatal androgen exposure provide evidence of organizational changes caused by steroid hormones during critical developmental periods in the sheep (61–63). Such critical periods in the sheep span from days 30 to 90 of gestation (63). More specifically, prenatal exposure to testosterone between gestational days 40 to 50 have been found to result in complete masculinization of the external genitalia with exposure a few days before and after also having an impact, albeit with a less severe impact (62). Urination pattern has also been shown to be affected by prenatal androgenization. Ewes exposed before day 90 and after day 30 of gestation show a male urination pattern (62). Sexual behavior of ewes prenatally androgenized also showed male-like mating behavior where they attempted to mount other ewes, with higher incidence among ewes prenatally exposed between days 50 to 100 and 70 to 120 (63). Early studies on ewes of prenatal androgen exposure also provide evidence of an altered neuroendocrine feedback system as discussed in the sections below (62).

The preoptic area is a sexually dimorphic region of the brain where majority of GnRH neurons are found. Differences in the size of this region between male and female rat brains was first reported by Gorski et al. in 1978 (64). Later studies confirmed the size differences between genders and termed that region of the medial preoptic area as the sexually dimorphic nucleus (SDN) (65). Subsequent studies were able to locate an analogous brain structure in sheep, which was termed ovine SDN (oSDN) (66). Interestingly, authors found that female-oriented rams had a larger oSDN compared to ewes and male-oriented rams. They also observed high aromatase mRNA expression within the female-oriented ram oSDN in comparison to that of the ewe and male-oriented rams (66). Further studies found that steroid hormones have an impact on the size of the oSDN. Female ovine fetuses exposed to prenatal testosterone between gestational days 60 to 90 had a masculinized oSDN. While prenatal exposure to testosterone during gestational days 30 to 90 had no impact on the size of the oSDN in the female fetus, these effects were in contrast to those seen in male fetuses. Prenatal androgenization between gestational days 60 to 90 had no impact on the size of the oSDN but exposure during 30 to 60 days of gestational life reduced the size of the male oSDN (67). Prenatal androgens can also have an impact on estrogen feedback mechanisms within the oSDN. Estrogen receptor α (ESR1) neurons are known to project to GnRH neurons in the preoptic area of the sheep brain (68, 69). Studies on prenatally androgenized ewes during the critical period of sexual differentiation show a disruption in estrogen and progesterone signals to the GnRH neurons evident by an altered LH response. The LH response of those same ewe’s mimic that of normal rams indicating the ability of prenatal androgens to masculinize the oSDN (70). Subsequent studies showed that estradiol-negative feedback disruptions are programmed specifically by androgen actions during prenatal exposure, while estradiol-positive feedback alterations occur largely due to the estrogenic effects after aromatization of androgens within the oSDN (71).

## 3 Neuroendocrine alterations in the sheep model of PCOS

**Figure 2:**
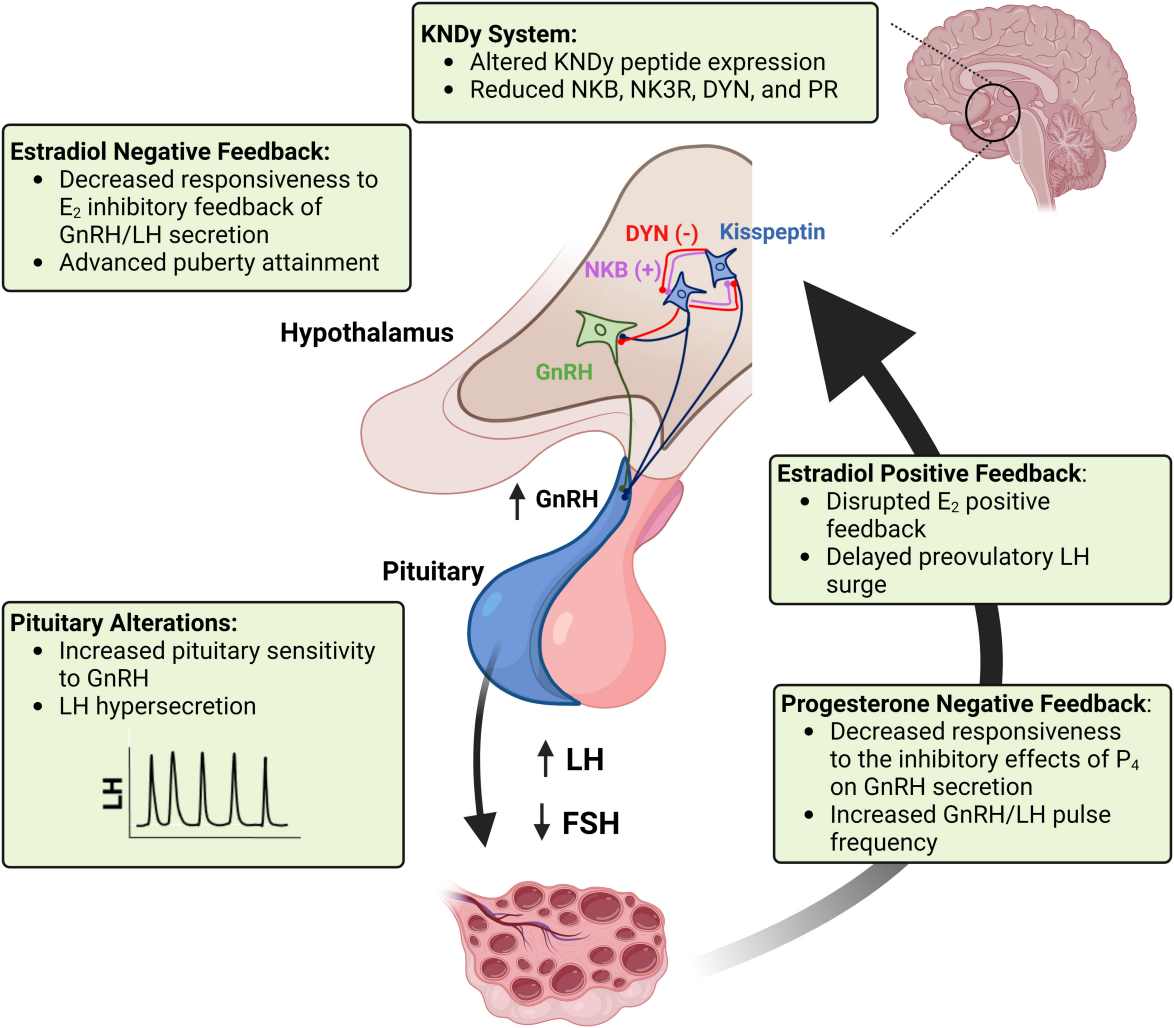
A schematic representation of the neuroendocrine alterations seen in the sheep model of PCOS phenotype. Imbalances in KNDy neuron peptide and receptor expression in the hypothalamus disrupt GnRH tonic secretion and responsiveness to steroid hormone feedback systems regulating reproductive cyclicity. Puberty attainment is altered due to decreased responsiveness to the estradiol (E2) inhibitory feedback. Reduced sensitivity to the progesterone (P4) negative feedback and increased pituitary sensitivity to GnRH result in LH hypersecretion and imbalance of the LH : FSH ratio, which in turn impairs follicular development and ovarian steroidogenesis. Impairments in the estradiol positive feedback mechanism result in disrupted (delayed and reduced amplitude) preovulatory surge of LH surge, thus compromising ovulatory capacity. Created with BioRender.com.

Prenatal testosterone-treated sheep exhibit neuroendocrine dysfunction similar to that observed in women with PCOS. Disruptions in the three steroid feedback systems that regulate reproductive cyclicity include reduced responsiveness to the estradiol negative feedback, estradiol positive feedback, and progesterone negative feedback (72). In ruminants, decreased responsiveness to the estradiol negative feedback and subsequent increased LH pulsatile secretion can advance sexual maturation in females (73). Defects in estradiol positive feedback mechanisms may alter important aspects of the preovulatory surge release of LH, such as timing and amplitude of the surge, thus impairing ovulatory capacity (44). Progesterone negative feedback alterations result in an increase in LH pulse frequency during the luteal phase, which in turn contributes to the development of functional hyperandrogenism and persistent ovarian follicles (74). In addition to alterations at the hypothalamic level, prenatal testosterone-treated sheep exhibit an increased pituitary responsiveness to GnRH stimulation (47, 75), further contributing to the development of LH excess and consequent functional hyperandrogenism in this sheep model of PCOS phenotype. These neuroendocrine alterations and the potential cellular and molecular mechanisms involved are discussed below and summarized in **Figure 2**.

### 3.1 Puberty and the estradiol negative feedback

Similar to humans and other mammalian species, the final developmental processes underlying sexual maturation in female sheep lie within the hypothalamus (76–78). During pubertal development, low-frequency secretion of GnRH pulses into the portal vasculature ultimately transitions into a higher-frequency mode of GnRH release. A corresponding increase in LH pulse secretion by gonadotrophs in the anterior pituitary follows and provides gonadotropic support for the final maturation of antral follicles, which in turn produce higher levels of estradiol to trigger first ovulation (36). The pulsative pattern of LH secretion is directly correlated with the synchronous depolarization of GnRH neurons (79). The process of synchronous depolarization of GnRH neurons is referred as the GnRH pulse generator (80, 81) and the cellular mechanisms controlling this process have begun to be elucidated in the past few decades after the discovery of kisspeptin. Disruption of kisspeptin signaling due to loss-of-function mutations on the genes encoding for kisspeptin or the kisspeptin receptor (Kiss1R) markedly impair GnRH secretion and prevent puberty attainment (82–84). Accordingly, administration of kisspeptin advances puberty in female rats (85) and sheep (86). From a neuroanatomical standpoint, a cluster of neurons within the arcuate nucleus (ARC) co-localizes three neuropeptides, kisspeptin, neurokinin B and dynorphin and have been termed KNDy neurons (87, 88). KNDy neurons release kisspeptin in response to neurokinin B stimulation (autocrine and paracrine), which in turn results in kisspeptin stimulation of GnRH cell bodies and GnRH neuronal projections (termed dendrons) to the median eminence of the hypothalamus (87, 88). Kisspeptin acts as the output signal from KNDy neurons to induce the synchronous depolarization of GnRH neurons. The depolarization of KNDy neurons promotes the release of dynorphin, which in turn hyperpolarizes and suppresses KNDy neuron activity. The sequential release of these three neuropeptides by KNDy neurons provides a likely mechanism underpinning the tonic release of GnRH (87, 89). Importantly, KNDy neurons express estrogen receptor-α (ESR1) (90) and estradiol suppresses *Kiss1* mRNA expression in the ARC (90, 91). During the early prepubertal stages, small amounts of estradiol produced by the ovarian follicles significantly suppress LH pulse frequency due to an increased hypothalamic sensitivity to the negative feedback effects of estradiol (36). However, during pubertal transition, the hypothalamus becomes less responsive to the estradiol negative feedback, thus resulting in increased pulsative secretion of GnRH/LH and gonadotropic support for follicular development (36). The increased LH pulse frequency during pubertal maturation provides gonadotropic support for the development of a preovulatory follicle with increased steroidogenic capacity. The resulting elevation in estradiol concentrations ultimately initiates a GnRH-induced surge of LH that triggers first ovulation.

Female sheep prenatally exposed to androgen excess between gestational days 30 to 90 have an advanced pubertal rise in LH secretion by approximately 10 weeks, which corresponds to the time of puberty in male lambs (92). Prenatal testosterone treatment markedly advanced pubertal onset in ewe lambs (22.5 weeks of age) compared to control females (27.5 weeks of age) (93). It is believed that the effects of prenatal androgen treatment advancing puberty are mediated by androgenic actions of testosterone, since prenatal treatment with dihydrotestosterone (DHT), a non-aromatizable androgen, also advances neuroendocrine puberty (92). This premise is supported by the observation that prenatal co-treatment with flutamide, an androgen antagonist, prevents the defeminization of the timing of puberty induced by prenatal testosterone treatment (93). The observation that prenatal testosterone treatment between gestational days 60 to 90 does not advance puberty in female sheep (44) suggests that the period between 30 to 60 days of fetal development is a susceptibility window for the programming effects of testosterone on timing of puberty.

From a mechanistic standpoint, prenatal exposure to testosterone excess in the 30-90 model reduced the responsiveness of the neuroendocrine system to the estradiol inhibitory effects on GnRH/LH secretion in 12-week old ewe lambs (juvenile) (43). This was characterized by an increased LH pulse frequency (3 pulses in 6 hours) after estradiol treatment compared to control females (1 pulse in 6 hours) (43). The effects of prenatal testosterone treatment reducing the responsiveness to the estradiol negative feedback are also manifested in a similar manner at 24 weeks of age when females are approaching puberty attainment (43). Collectively, these studies provide evidence that an early reduction in the neuroendocrine sensitivity to the estradiol negative feedback on GnRH/LH secretion is expressed before puberty in prenatal testosterone females, similar to observed in hyperandrogenic girls prior to menarche (11, 12). The effects of prenatal testosterone disrupting the estradiol negative feedback are also likely programmed by the androgenic actions of testosterone, since both testosterone and DHT, but not co-treatment of testosterone and flutamide, reduced the sensitivity to the inhibitory effects of estradiol on LH secretion (43, 71). Because KNDy neurons represent a critical neuronal pathway by which gonadal steroids regulate GnRH pulsatile secretion (87, 88), neuropeptide imbalances within these neurons could underlie this neuroendocrine defect in prenatal testosterone sheep (94). The neuropeptide and receptor imbalances within KNDy neurons observed in this sheep model are discussed below under the “*Progesterone Negative Feedback and LH Hypersecretion*” section. Additionally, prenatal testosterone-treated sheep exhibit elevated circulating concentrations of leptin, a hormone produced by the adipose tissue that signals energy status to the brain (95). Because leptin accelerates GnRH pulsatility and controls puberty (96), it is possible that increased levels of leptin reduce the hypothalamic sensitivity to the estradiol negative feedback and advance the pubertal rise in LH secretion in this sheep model.

### 3.2 Estradiol positive feedback and the LH preovulatory surge

During the late-follicular phase, increased levels of estradiol produced by the preovulatory follicle reach a threshold required to activate the estradiol positive feedback and subsequently initiate the preovulatory surge release of GnRH and LH (97). The LH surge is not only a critical process that triggers ovulation, but it also induces maturation of the oocyte and the conversion of the follicle into progesterone-producing luteal tissue (98). The amplitude and duration of the preovulatory surge of LH may affect subsequent luteal development and function, since it has been shown that females that ovulate after a larger LH surge (total LH released during the surge) had a significantly earlier rise in postovulatory concentrations of progesterone compared to those with a smaller surge of LH (98, 99).

The preovulatory surge of LH is delayed and the magnitude of the surge is significantly reduced in prenatal testosterone treated females. Both models of prenatal testosterone treatment, the D30-90 and D60-90, demonstrate delayed responses to the estradiol positive feedback, which are characterized by a delayed onset of the LH and FSH surges (44). Although both models exhibit delayed surges, the delay is more pronounced in the D30–90 than in the D60–90 females (44), suggesting that the earlier window between 30 to 60 days of fetal development is important programming this neuroendocrine defect. In addition to the timing of the surge, the amplitude of the LH surge was reduced in GD30-90 testosterone-treated ewes (44). In GD30-90 testosterone-treated ewes, 64% of the animals showed reproductive cycle, however, only 6% demonstrated normal responsiveness to the estradiol positive feedback (44). Sheep prenatally treated with testosterone between gestational days 30-90 exhibited LH surges of lower magnitude compared to control females (44, 100). These observations provide clear evidence that prenatal exposure to androgen excess disrupts the estradiol positive feedback mechanism required to generate the preovulatory surge release of GnRH and LH. This impairment in the estradiol positive feedback mechanism, characterized by delayed and low-magnitude surges of LH and FSH, may result in ovulatory defects. The effects of prenatal exposure to androgen excess disrupting ovulatory capability and normal estrous cyclicity in sheep have been reported previously (70, 101).

There are two kisspeptin cell populations found in the ovine hypothalamus, one in the preoptic area and another in the ARC. Kisspeptin neurons in the preoptic area do not coexpress dynorphin or neurokinin B unlike the ARC kisspeptin neurons (102). In rodents, the anteroventral periventricular nucleus has been shown to be the primary hypothalamic area mediating the estradiol positive feedback effects on LH secretion (19, 103–105). However, in sheep the primary site of the surge-generating mechanism is likely the caudal region of the ARC (106, 107). Caraty et al. in 1998 showed that placement of estradiol implants into the medial basal hypothalamus, but not in the preoptic area, is able to induce a GnRH surge in the ewe (106). Also in sheep, it has been demonstrated that kisspeptin expression in the preoptic area and ARC are sexually dimorphic with more kisspeptin cells found in females than males for both hypothalamic sites (94). Notably, prenatal testosterone treatment between gestational days 30-90 did not alter the average number of kisspeptin-positive cells in the preoptic area or the ARC (94). However, prenatal exposure to androgen excess markedly reduced the numbers of neurons expressing the neurokinin B receptor NK3R in the ARC in sheep (108). Importantly, this reduction in NK3R expression was mainly a result of changes within KNDy neurons and not in other neuronal populations in the ARC (108). Additionally, prenatal testosterone-treatment decreased the number of cells expressing neurokinin B in the ARC (94) and it has been postulated that the combined reduction in both ligand and receptor may contribute to defects in the estradiol positive feedback and the LH surge mechanism in this model (108). The involvement of the neurokinin B system in the preovulatory LH surge in sheep is supported by the observations that KNDy neurons express cFos, a marker of neuronal activity, during the preovulatory surge of LH (109, 110) and that central injections of a neurokinin B agonist induce activation of KNDy neurons and elicit a surge-like release of LH (111). Because central administration of kisspeptin antagonist only reduces the preovulatory LH surge amplitude by 50% in sheep (112), it is conceivable that kisspeptin and neurokinin B act synergistically to stimulate LH secretion during the preovulatory surge (108). Taken together, these observations suggest that neurokinin B signaling plays an important role on the estradiol positive loop that triggers the preovulatory LH surge and that alterations within the neurokinin B system (peptide and receptor) may disrupt this neuroendocrine feedback mechanism in prenatal testosterone-treated sheep.

### 3.3 Progesterone negative feedback and LH hypersecretion

During the menstrual cycle in women and the estrous cycle in sheep, progesterone produced by the corpus luteum suppresses GnRH neuron activity *via* a negative feedback system. This classic neuroendocrine feedback mechanism between the ovaries and the hypothalamus is impaired in women with PCOS (7, 113), resulting in hyperactivity of GnRH neurons, which in turn results in elevated LH pulse frequency and amplitude, elevated serum concentrations of LH, and an increased LH : FSH ratio (113–115). The increased episodic release of LH leads to increased androgen synthesis by theca cells and systemic hyperandrogenism, a hallmark of PCOS (116, 117). Interestingly, evidence from clinical and preclinical studies suggest that hyperandrogenism acts within the brain to drive the hypersecretion of GnRH and LH, thus creating a vicious cycle of androgen excess and impaired neuroendocrine function (113). However, androgens do not stimulate pulsatile secretion of LH directly, evidenced by the fact that testosterone administration does not increase LH episodic release in PCOS women (9) and acute administration of an androgen antagonist does not restore secretion of LH to normal levels in PCOS women (10). Rather, evidence suggests that androgen excess impairs the ability of progesterone to act within the hypothalamus and suppress GnRH secretion, therefore contributing to the development of GnRH/LH hypersecretion (113). This premise is supported by the observations that women with PCOS require higher amounts of progesterone to suppress LH secretion to similar levels as seen in healthy women (7) and that long-term treatment with an androgen antagonist restores progesterone’s ability to suppress LH secretion in women with PCOS (10).

Similar to observed in women with PCOS, prenatal testosterone-treated sheep exhibit impairments in the responsiveness of the neuroendocrine system to the progesterone negative feedback. Robinson and colleagues (118) performed studies using ovariectomized sheep to determine whether the inhibitory actions of progesterone on LH pulse frequency are sexually dimorphic and affected by prenatal exposure to androgen excess. Authors observed that while exogenous progesterone administration markedly inhibited LH pulse frequency in control females, progesterone treatment had no effect on mean LH concentrations, pulse frequency, or pulse amplitude in males and prenatal testosterone-treated female sheep (118). Notably, this reduced responsiveness to the progesterone feedback mechanism was observed in female sheep prenatally treated with testosterone during either gestational days 30 to 90 or 60 to 90, suggesting that the shorter developmental window between 60 and 90 days of gestation is sufficient to program this neuroendocrine defect. Similarly, mean LH concentrations and LH pulse frequency during the mid-luteal phase were significantly higher in prenatal testosterone-treated females than in controls, despite similar circulating concentrations of progesterone, confirming the ability of prenatal androgen excess to decrease the responsiveness of the neuroendocrine system to the negative feedback effects of progesterone (94).

GnRH neurons do not contain progesterone receptors, suggesting that progesterone negative feedback on GnRH release is not transduced through GnRH neurons (119), but rather through afferent systems. KNDy neurons colocalize progesterone receptor and have been shown to mediate progesterone negative feedback response on GnRH secretion (94). The progesterone receptor was found to be colocalized in more than 90% of parvicellular dynorphin neurons in the preoptic area, ARC, and anterior hypothalamus (120). Dynorphin, an inhibitory peptide, mediates progesterone negative feedback of GnRH pulse frequency during the luteal phase of the estrous cycle in the sheep model (121). Microimplants of progesterone receptor antagonist RU486 into the ARC disrupted progesterone negative feedback on LH pulse frequency in sheep (122). Gestational testosterone treatment reduced the number of dynorphin (94, 123) and progesterone receptor-positive cells (94, 123), but kisspeptin remained unchanged (94) in the ARC of female sheep. These divergent effects of prenatal testosterone on dynorphin and kisspeptin expression led to the hypothesis that the decrease responsiveness to the progesterone negative feedback is due to changes in the balance of neuropeptide abundance within KNDy neurons (94). A reduction in inhibitory neuropeptide expression (dynorphin) coupled with no changes in stimulatory peptide (kisspeptin) may shift the net balance of peptidergic input to GnRH neurons toward the excitatory side and reduce the capacity of progesterone to inhibit episodic release of GnRH in prenatal testosterone-treated sheep (94). This neuroendocrine disruption may be further exacerbated by the fact that prenatal androgen excess decreases the number of progesterone receptor-containing neurons in the ARC of the hypothalamus (124).

### 3.4 Pituitary alterations and GnRH hyperresponsiveness

In addition to hypothalamic alterations, the anterior pituitary also contributes to the development of LH hypersecretion in PCOS (13, 125). Women with PCOS exhibit LH responses to exogenous GnRH administration greater in magnitude than those observed in healthy women during the follicular phase of the menstrual cycle (14, 125, 126). Interestingly, the circadian changes in the LH and insulin circulating concentrations in women with PCOS follow a similar time course (127), suggesting a positive correlation between insulin and LH. A potential role of insulin in increasing pituitary sensitivity to GnRH is supported by the observation that treatment with rosiglitazone, an insulin sensitizer, not only improves peripheral insulin sensitivity, but also reduces the LH concentrations in women with PCOS (128, 129).

Similar to PCOS women, prenatal testosterone-treated sheep exhibit increased pituitary responsiveness to GnRH stimulation, characterized by a marked increase in LH pulse amplitude following exogenous GnRH treatment (47, 75). Recent research investigated the effects of administration of either flutamide (androgen antagonist) or rosiglitazone (an insulin sensitizer) on GnRH-stimulated LH secretion in prenatal testosterone sheep (47). In this study, prenatal testosterone-treated sheep demonstrated greater LH pulse amplitude and pulse peak compared to controls (47). Importantly, insulin sensitizer treatment restored the amplitude of GnRH-stimulated LH pulses to control levels, suggesting that this neuroendocrine defect is largely secondary to perturbations in insulin-glucose homeostasis as reported in PCOS women. At the tissue level, prenatal testosterone increased pituitary protein levels of LHβ while it markedly decreased estrogen receptor (ESR1) protein levels in adult sheep (47). As mentioned previously, prenatal testosterone-treated sheep have reduced neuroendocrine responsiveness to the inhibitory effects of estradiol on LH secretion (43). While this regulatory mechanism occurs largely at the hypothalamic level, observations in the female sheep suggest a direct pituitary effect (130). Studies in hypothalamo-pituitary disconnected sheep observed a decrease in the amplitude of GnRH-stimulated LH pulses following administration of estrogen (130, 131). Therefore, the finding that prenatal androgen excess diminishes the pituitary protein expression of ESR1 in adult sheep suggests that the reduced sensitivity to the estradiol inhibitory effects on LH secretion is partially mediated at the pituitary level. Moreover, observations that rosiglitazone treatment restored pituitary ESR1 expression to normal levels indicate that this may be one mechanism by which insulin sensitizer treatment prevents LH hypersecretion in this sheep model. Studies using fetal pituitaries indicate that programming of pituitary dysfunction starts during prenatal development and continues until adulthood in prenatal testosterone sheep, since several alterations in protein expression can be detected early during fetal development (132).

## 4 Conclusions and translational relevance

Neuroendocrine imbalances, such as increased pulsatile secretion of GnRH and enhanced pituitary sensitivity to GnRH stimulation, contribute to the etiology of PCOS. LH hypersecretion promotes ovarian hyperandrogenism, which in turn impairs the responsiveness of the neuroendocrine system to the progesterone and estradiol negative feedback mechanisms, thus creating a vicious cycle between LH hypersecretion and hyperandrogenism (5). Prenatal testosterone-treated sheep recapitulate the reproductive and metabolic phenotypes of PCOS and provide unparalleled resources to investigate cellular and molecular changes leading to neuroendocrine dysfunction. Prenatal testosterone-treated sheep exhibit alterations in all three steroid feedback mechanisms controlling GnRH/LH secretion, namely estradiol negative (43), estradiol positive (44), and progesterone negative feedback (45, 46). Furthermore, pituitary sensitivity to GnRH is markedly increased in these animals (47). At the hypothalamic level, imbalances in peptide abundance and receptor expression within the KNDy system are likely key alterations leading to GnRH neuron hyperactivity. At the anterior pituitary level, peripheral hyperinsulinemia may contribute to the GnRH-stimulated LH hypersecretion and restoration of pituitary ESR1 expression is likely a mechanism by which insulin sensitizer treatment prevents LH hypersecretion in prenatal testosterone sheep.

Considering the likely role of KNDy neurons in the PCOS pathogenesis, the KNDy signaling system represents a critical therapeutic target for treatment of neuroendocrine dysfunction in women with PCOS (113). Recent clinical studies reported that treatment with AZD4901, a neurokinin-3 receptor antagonist, specifically reduced LH pulse frequency and subsequent serum concentrations of LH and testosterone in PCOS women (133). Additionally, treatment with naltrexone, an opioid receptor antagonist, resulted in weight loss and reduced LH concentrations and LH : FSH ratio in women with PCOS (134). Moreover, co-treatment with pulsatile GnRH and naltrexone improved ovulation induction outcomes compared to GnRH alone in obese women with PCOS (135). Due to the complexity and heterogeneity of PCOS phenotypes, additional therapies are needed. Animal models of PCOS, including the sheep model, can aid with the identification of novel therapeutic targets for treatment and prevention of neuroendocrine dysfunction in women with PCOS and other hyperandrogenic conditions.

## Author contributions

All authors listed have made a substantial, direct, and intellectual contribution to the work and approved it for publication.
